# A Retrospective Study of Orthoptic Students’ and Teaching Experience with the Introduction of Technology Promoting a Blended Learning Environment: *Experiences with technology in a blended learning environment*

**DOI:** 10.22599/bioj.119

**Published:** 2018-10-09

**Authors:** Carla Lança, Anne Bjerre

**Affiliations:** 1 Escola Superior de Tecnologia da Saúde de Lisboa, Instituto Politécnico de Lisboa, PT; 2 University of Sheffield, GB; 3 Singapore Eye Research Institute, SG

**Keywords:** blended learning, COLLES, moodle platform, orthoptic program

## Abstract

**Purpose:**

Evaluation of the students’ experience and academic achievements following the introduction of online learning.

**Methods and materials:**

In 2011, online learning activities were introduced in the teaching of the Research in Orthoptics module for final year undergraduate orthoptic students. The online learning activities were created and delivered in Moodle; an open-source online learning platform. Students from the academic year groups of 2012–13, 2013–14 and 2016–17 completed an online questionnaire. The questionnaire was divided into 6 categories (relevance, reflection, interactivity, tutor support, peer support and interpretation) with 4 questions within each category. A 5-point Likert scale was used to score each question. The sum of answers within each category ranged from 4 (negative perception) to 20 (positive perception). Student performance was assessed using the marks retrospectively for 2 years before online learning was introduced and when online learning was included.

**Results:**

Forty-two students replied to the questionnaire with a mean age of 23.0 ± 2.3 years (range 21–32). There were 38 females (90.5%) and 4 males (9.5%). Combining the 3 academic cohorts there was a significant difference between the 6 categories of the questionnaire (p < 0.0001). Three categories obtained the same high median score of 16: relevance (range 9–20), reflection (range 11–20) and tutor support (range 12–20). Peer support resulted in the lowest median score of 13. Separation of the three academic year cohorts’ revealed significant differences for tutor support (p = 0.03). The score increased from 16 in 2012–13 and 2013–14 to 18 in 2016–17. Significant differences were found between the marks for the cohorts from 2009–10 to 2016–17 (F_7,245_ = 5.07: p < 0.0001). The mean mark for year group 2009–2010 was significantly less compared to 2012–13 (p < 0.0001), 2014–15 (p = 0.01) and 2015–16 (p = 0.02) and year group 2011–12’s mark significantly less than 2012/13 (p = 0.001).

**Conclusions:**

Including online learning in the research module had a positive impact on the student experience, although more work needs to be done to improve peer support. Marks obtained by the orthoptic students have improved since the introduction of online learning suggesting that a mixture of teaching and learning methods is beneficial for students. However, more work needs to be done to provide teacher assistance in the design of online learning and blended learning approach.

## Introduction

Advances in knowledge have resulted in an increasing use of technology for personal and educational purposes. Online learning can be defined as educational material presented on a computer (Carliner, 1999) and is an innovative approach to delivering instructions to a remote audience using the web as the medium (Khan, 1997). Online learning requires the learner to use the internet to access learning materials; interact with the contents, tutor and other learners; and to obtain support during the learning process (Ally, 2004). A range of online learning management software systems is available for educators such as Moodle, Blackboard, and Canvas. The use of online learning is growing in medical education (Ellaway and Masters, 2008). The online learning environment refers to a computer-mediated space in which online distance education occurs. The reasoning for introducing online learning as one of the pedagogic approaches of teaching is manifold. Students are generally computer and digital literate and have easy access to computers and other digital devices such as mobile phones and tablets. There is also a growing demand for online learning at a time convenient to the students (Khogali *et al*., 2011).

Currently, incorporating technology is one of the major goals within learning, teaching and digital strategy in higher education institutions. (The University of Sheffield, 2016; University of Oxford, 2017). A further goal is for academic educators to provide more efficient, effective and innovative learning and teaching methods. Providing an online learning platform with a range of resources such as lecture handouts, videos, web links and online activities may increase learning potential (McBrien, Jones and Cheng, 2009). The benefit of including online learning such as self-directed and group-based learning is that the students are encouraged to be active learners and can do the activities at their own pace. However, students have identified that combining online learning and face-to-face teaching is more beneficial than solely online learning (Morton *et al*., 2016).

Blended learning refers to a mixture of different pedagogical approaches such as online learning combined with face-to-face engagement in the form of lectures and small group tutorials (Garrison and Kanuka, 2004). It has been reported as more effective, as that approach combines a range of possibilities to engage students using technology and distance education with classroom forms and face-to-face instruction resulting in higher levels of student engagement. Although e-learning is an effective approach in many medical schools, it should not replace traditional learning (Makhdoom *et al*., 2013). Blended learning is a better approach using a balanced methodology with the advantages of technology and the benefits of face-to-face learning.

Studies evaluating the use of blended learning have shown it can potentially improve healthcare students’ clinical competencies (Rowe, Frantz and Bozalek, 2012), increase student engagement (Lewin *et al*., 2009) and medical students have demonstrated statistically significant better performance in exams compared to students receiving traditional teaching without any online learning material (Makhdoom *et al*., 2013). A systematic review and meta-analysis of 56 studies evaluating blended learning in health professional learners supports that blended learning can have a positive effect on knowledge acquisition compared to non-blended learning, and is just as effective as the non-blended approach (Liu *et al*., 2016). Although there are a number of studies that have explored the benefits of blended learning for students studying medicine (e.g. ophthalmology) (Iyeyasu *et al*., 2013) and some other eye healthcare subjects (optometry) (Gupta and Gupta, 2016), the benefit for students studying orthoptics has not been reported. Furthermore, to our knowledge the impact of long-term implementation of blended learning for students studying healthcare subjects has not been reported. A vast number of orthoptic training institutions are already using varying levels of information and communication technologies (ICT) based teaching and learning practices, however sustainable use of technology and ability to electronically access and analyze data is still in its infancy (Gupta and Gupta, 2016).

At Lisbon School of Health Technology, fourth-year undergraduate orthoptic students are off-campus for a significant part of the semester, undertaking professional practice experiences (clinical placement) at eye clinics in hospitals, primary care centers or in private sponsored practices across Portugal. In 2011, the use of online learning and a blended learning approach was introduced into the Research in Orthoptics module. The main reason for the implementation of this teaching approach was to enable students to perform group work while on clinical placement, but also to enhance the students’ learning experiences. While this approach has been rolled out across Portuguese higher education institutions in the last few years, there are few studies of how individual disciplines have implemented these approaches and what challenges they have faced. Evaluation of the students’ experiences and any change in academic achievements following the introduction of online learning and a blended learning approach was analyzed. This study evaluated the student feedback and assessment outcomes of final year orthoptic students completing the Research in Orthoptics module. The aim was to determine the orthoptic students’ perception of using an online learning environment and to compare across different year groups. In addition, any change in overall assessment mark was determined.

## Materials and Methods

### Learning strategies

In 2011–12, online learning activities were introduced in the teaching of the Research in Orthoptics module for final year undergraduate orthoptic students (4-year program) at Lisbon School of Health Technology (ESTeSL), Portugal. As students went on clinical placement during the final year, the introduction of online learning activities was carried out to comply with innovate learning approaches used by ESTeSL and also to diminish students travelling to the school.

The same lecturer delivered the teaching of this module in the period from 2009–10 to 2016–17. Teaching approaches were as follows:

- Between 2009–10 to 2010–11, students were taught using face-to-face learning with traditional lectures. Students had a total of 3 hours face-to-face teaching per week for a total of 15 weeks.
- In 2011–12, a blended learning approach was introduced. Face-to-face learning with traditional lectures alternated with online learning activities (rotation cycle) every 15 days. Students had a total of 3 hours face-to-face teaching or online learning activities per week for a total of 15 weeks.

The online learning activities were created and delivered in Moodle (Modular Object-Oriented Dynamic Learning Environment), which is a free open-source online learning platform. The lecturer received training for the use of Moodle during the academic year of 2010–11. Students were introduced and prepared for the e-learning environment with an hour long face-to-face presentation by the lecturer. Students also received a written PDF tutorial with an explanation of the online activities. The lecturer provided support throughout the module when requested by the students.

The face-to-face teaching at the university on alternate weeks changed from the traditional didactic lecture style to small group work and class discussion activities. For the online activities, students were required to work independently but also to participate in asynchronous online group work. The amount of online activities was increased over time according to the lecturer’s familiarity with the online component as follows:

1. In the first and second years of implementation (2011–12 and 2012–13), students had access to handouts from lecture presentations and scientific papers (additional readings). Web links and videos were also added. Students had to engage in group activities to prepare a summary to share their views about the online content when in class.
2. In the following years (2012–13 to 2016–17), online alerts (to inform students about new online activities) and interactions between the lecturer and students (e-mails sent through the online platform for doubts and questions) were used facilitating communication and collaboration.

### Marks

Marks were analyzed to determine the potential influence of online learning and blended learning approach on student performance. The overall group mark for the summative assessment was evaluated retrospectively for 2 years before online learning was introduced and from 2011–12 to 2016–17 when online learning was included. Summative assessment was comprised of a group assignment after 8 weeks of teaching (accounted for 20% of the total mark), an independent written assessment at the end of the module (accounted for 40% of the total mark), and one month after completion of the module, each student submitted a research project proposal (accounted for 40% of the total mark). All the written assessments were submitted online. The marking scale was defined as a mark of 18–20 (excellent), 16–17 (very good), 14–15 (good), 10–12 (sufficient), and <10 (fail). This scale was applied to all assignments.

### Questionnaire

To evaluate the student experience in the Research in Orthoptics module, students from the academic year groups of 2011–12, 2012–13, 2013–14 and 2016–17 were invited to complete an online survey anonymously. The 2016–17 group was included to determine if the student perspective had changed as technology had advanced and students were more familiar using technology both for personal and education use. No data was collected in 2014–15 and 2015–16.

The Moodle platform contains survey activities of three types of pre-made, standard and verifiable survey instruments that help the lecturer to understand what students are thinking. The actual form of Constructivist Online Learning Environment Survey (COLLES) developed by Taylor and Maor (2000) to examine students’ perceptions of using an online learning environment was used for this study. The COLLES has 24 statements grouped into six scales (relevance, reflection, interactivity, tutor support, peer support and interpretation), each of which help to assess the quality of the online learning with four questions within each scale (Table 1).

**Table 1:** Displays all the questions and categories for the Constructivist Online Learning Environment Survey (COLLES) (Taylor and Maor, 2000) used in this study.

Question(Q)/Category		Q1.	Q2.	Q3.	Q4.	Response rate: 1 – almost never to 5 – almost always. Combined score

1.	Relevance	My learning focuses on issues that interest me.	What I learn is important for my professional practice.	I learn how to improve my professional practice.	What I learn connects well with my professional practice.	4–20
2.	Reflection	I think critically about how I learn.	I think critically about my own ideas.	I think critically about other students’ ideas.	I think critically about ideas in the readings.	4–20
3.	Interactivity	I explain my ideas to other students.	I ask other students to explain their ideas.	Other students ask me to explain my ideas.	Other students respond to my ideas.	4–20
4.	Tutor support	The tutor stimulates my thinking.	The tutor encourages me to participate.	The tutor models good discourse.	The tutor models critical self-reflection.	4–20
5.	Peer support	Other students encourage my participation.	Other students praise my contribution.	Other students value my contribution.	Other students empathies with my struggle to learn.	4–20
6.	Interpretation	I make good sense of other students’ messages.	Other students make good sense of my messages.	I make good sense of the tutor’s messages.	The tutor makes good sense of my messages.	4–20

A 5-point Likert scale was employed to score the answer to each question on the questionnaire (1 – almost never to 5 – almost always). The sum of the 4 answers to each question within the 6 categories ranged between 4 (negative perception) and 20 (positive perception).

The COLLES questionnaire took approximately 10–15 minutes to complete. Prior to completing the survey, participants were informed that the information given would be anonymous and confidential (a code number was assigned to each participant). Also stressed was the voluntary nature of the survey, and that participants should only complete the questionnaire if they consented for their answers to be evaluated. This study adhered to the tenets of the Declaration of Helsinki.

### Statistical analysis

The responses from the COLLES questionnaire were statistically analysed using GraphPad Prisms, version 7 (GraphPad software, UA). As the data was ordinal, non-parametric analysis was performed. Descriptive analysis comprised of calculating the median and inter-quartile range. To determine if there was a significant difference in overall score between the six categories a Friedman test was performed. Kruskal-Wallis analysis was conducted to establish any significant difference in the score between the academic year groups for each category.

The mean, standard deviation and range data for each year groups’ summative assessment were calculated. To determine if there was a significant difference between the year groups one-factor ANOVA and post-hoc Bonferroni’s multiple comparison tests were performed.

## Results

Twenty-one students participated in the research module in 2011–12, 35 in 2012–13, 36 in 2013–14 and 24 in 2016–17 (total = 116). A total of 45 students completed the questionnaire from the four academic year cohorts: 3 (21%) students from 2011–12, 12 (34%) students from 2012–13, 9 (24%) from 2013/14 and 21 (84%) from the 2016/17 cohort. Due to the very small completion rate for the 2011–12 cohort, researchers decided to exclude that data in the analysis and only present the findings for the 2012–13, 2013–14 and 2016–17 cohorts. The mean age of the 42 students were 23.0 ± 2.3 years (range 21–32). There were 38 females (90.5%) and 4 males (9.5%).

**Figure 1:**
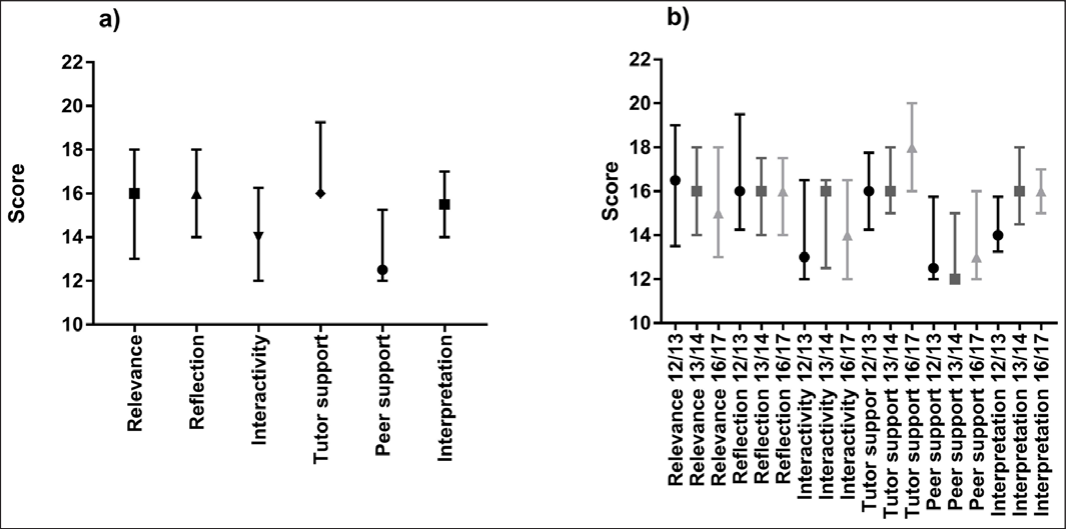
Shows the median and interquartile range scores for the six categories on the COLLES questionnaire: **a)** Combined scores of all three-year groups (2012/13, 2013/14 and 2016/17); **b)** Separate scores for each year group.

The median and interquartile range data combining the 3 academic cohorts and separating into academic years are shown in Figure 1a and 1b respectively.

Combining the three academic cohorts, Friedman analysis demonstrated a significant difference between the six categories (p < 0.0001). Three categories obtained the same high median score of 16; these were relevance (range 9–20), reflection (range 11–20) and tutor support (range 12–20). Peer support resulted in the lowest median score of 13 and the largest range of 4–20.

Separating the three academic year cohorts’ category data, Figure 1b demonstrates that the median values only differ within three scores for each category. No significant difference was found between the three academic cohorts for each category (p > 0.05) except for tutor support (p = 0.03) where the score increased from 16 in 2012–13 and 2013–14 to 18 in 2016–17 (Kruskal-Wallis analysis). Although not statically significant, peer support and interpretation scores increased slightly over time and relevance gradually decreased from 16.5 in 2012–13 to 15 in 2016–17. Reflection remained the same with a median score of 16 for all three cohorts.

Table 2 shows the average summative assessment mark for each year group completing the Research in Orthoptics module. Year group 2009–10 and 2010–11 received teaching without the blended learning approach. The teaching for year group 2011–12 to 2016–17 was a blended learning approach. The same lecturer taught this module from 2009–10 to 2016–17.

**Table 2:** Shows the mean, standard deviation and range of summative assessment marks for each year group from 2009–10 to 2016–17.

	2009/10	2010/11	2011/12	2012/13	2013/14	2014/15	2015/16	2016/17

**No of students**	31	30	21	35	36	42	34	24
**Mean**	13,87	15,20	13,95	16,03	15,06	15,43	15,44	14,75
**SD**	1,60	2,23	1,89	1,48	1,73	2,04	1,59	1,48
**Min**	12	10	11	12	12	11	12	13
**Max**	16	18	17	18	17	20	19	18

The spread of marks ranged between 10 (categorised as sufficient) to 20 (categorised as excellent) and the mean marks between 13.87 (sufficient) and 16.03 (very good). One-way ANOVA showed a significant difference between the year groups (F_7,245_ = 5.07: p < 0.0001). Post-hoc Bonferonni’s multiple comparisons test revealed the year group marks that were significantly different from each other. The mean mark for year group 2009–10 was significantly less compared to 2012–13 (p < 0.0001), 2014/15 (p = 0.01), 2015/16 (p = 0.02) and year group 2011–12’s mark significantly less than 2012–13 (p = 0.001). Comparison of the remaining year group marks were not significantly different (p > 0.05).

## Discussion

In this study the results of combining the 3 academic cohorts showed that students rated participation in online learning the highest on the aspects of professional relevance (importance of research for professional practice), reflection (critical thinking) and tutor support. In a similar study in pharmacy education, students rated professional relevance the highest and interactivity (making good sense of tutor and student’s messages) the lowest (Sthapornnanon *et al*., 2009). Although not significant, in the present study there was a decrease in the category relevance over time. This result shows that the students might perceive research as less relevant to the orthoptic professional practice comparing to other subjects. After graduation, the newly orthoptists might rate research as less relevant and there is the need to make research more appealing to orthoptic practice. Research results can take time to be diffused into orthoptic practice. There is a need to close the gap between research and orthoptic practice. This warrants further investigation about the increase in the awareness of the importance of research to their clinical practice.

The separate analysis for each academic cohort showed a significant difference in tutor support that increased over time. Rating the tutor support higher than peer support could reflect that the students are still very dependent on the lecturer (Syed-Mohamad *et al*., 2006). Other studies reported similar results (Taylor and Maor, 2000; Dougiamas and Taylor, 2002; Sthapornnanon *et al*., 2009), which may indicate difficulties in the transition from a passive to an active learning style. However, this result might also reflect the lecturer’s experience with blended learning approaches and online learning that increased over time. Students may have replied to the questionnaire based on their interaction with the lecturer in the face-to-face approach, rather than their online learning.

The lowest score was attributed to peer support, showing that one of the weaknesses of the online learning according to student’s perception is the lack of personal interaction between peers. Before technology was introduced the teaching relied on the lecturer with limited peer support opportunities. Students may have found the transition of carrying out peer support online difficult and more lecturer encouragement may have increased this interaction. A review of orthoptic student’s activities in the e-learning platform showed that the students did not use the e-learning environment in a systematic way (only a few interactions with peers) to discuss research related content. Orthoptic students used the online platform frequently to submit and exchange learning materials and relied mostly on learning materials submitted by the lecturer/tutor. In these cohorts the tutor did not systematically control this parameter, giving the students the opportunity to engage themselves online without supervision. This strategy may account for lower scores in peer support. These lower scores need to be analyzed carefully, as this may impact the newly graduate orthoptists behavior when doing teamwork. One of the main outcomes is that more work needs to be done, to reinforce student’s strategies to work collaboratively with their peers in the learning online environment, using peer learning systems. Adding to this, lecturer’s knowledge and education about peer support should be increased. Strategies to engage and encourage students to collaborate online should be used by the tutor. Students can be taught to improve peer support and develop skills in teaching, mentoring, organizing and managing. Peer assisted learning or peer teaching and learning is a collaborative pedagogical resource used in active participations with colleagues during discussions (Secomb, 2008). Meaningful and active online discussion among students will result in an effective knowledge sharing and cognitive development (Sthapornnanon *et al*., 2009). Forums of open discussion, moderated by the lecturer, throughout the orthoptic course need to be implemented. Also, students need to be taught how to interact effectively in the online learning environment (Wozniak, 2006). One option in online collaborative work is to ask students to share their interpretation of reading academic papers (MacDonald, 2008). According to Salmon, (Salmon, 2003) online learning offers more opportunities for students to write for themselves, which benefits both their own learning and each other’s. Adding to this, students may be asked to adopt the role of debate moderator to deepen their contribution in online communication. Based on the findings of the present study, future cohorts of 2017–2018 will receive a systematic online discussion between peers (e.g. online chats and forums) to improve peer support and enable more online engagement.

In the present study, summative assessment mark for each year group showed that the cohort from 2009–2010 differed significantly from the other cohorts exposed to online learning and a blended learning approach. By 2012–2013, the second year of the introduction of the online learning activities, there was a significant increase in the overall marks compared to when no online learning was included. However, the difference in mean mark between 2014 and 2017 was small and is not indicative of a continuous improvement in marks. The marks do not show a definite increase each year and this finding may be related to the way technology was introduced each year. Data before 2014 suggests a trend in improvement of student experience including online learning. Part of this improvement could be due to the increase in lecturer and students experience using technology. In 2012–13, the lectures became more interactive online. Curiously, this was the academic year where the students achieved higher marks. Also, the 2010–2011 cohort received teaching without the blended learning approach had slightly better marks comparing with the 2016–2017 cohort. This result might reflect an increase in teaching experience from year 1 to year 2. A previous study showed that the motivation generated by face-to-face activities is more explanatory of the final marks, (López-Pérez, Pérez-López and Rodríguez-Ariza, 2011) as the students have a higher degree of involvement in the learning process. Similar studies reported that a blended learning environment has positive impact in academic achievement (McLaughlin *et al*., 2015) and in final marks (López-Pérez, Pérez-López and Rodríguez-Ariza, 2011). One study shows that the level of Australian orthoptic student’s engagement with online discussion was found to be significantly correlated with the marks, particularly the practical examinations (Wozniak, 2006). However, motivation can be a factor that leads students to report higher perceptions of their learning. Students who are more reliant on instructor’s support tend to perceive their learning outcomes higher in a blended learning environment (Lo, 2010). Also, there are differences between students, with highest achievers being the most satisfied with a blended course (Owston, York and Murtha, 2013). On the contrary, the lowest achievers are least likely to want to take another blended course and prefer face-to-face instruction. Another factor is that there may be a difference between perceived and actual learning.

For the last four years the marks were fairly stable, with a slightly decrease in 2016–2017. This finding can be related to the increased students and lecturer experience with the online technology. For future cohorts, the mark might decrease if the teaching approaches do not change to support students’ increase knowledge in digital literacy. Although this finding supports the use of online learning activities in new cohorts of orthoptic students, it also supports that lecturer and student training is needed. Achievement of improved outcomes is a constant concern for lecturers, and a change in teaching methods might be required (López-Pérez, Pérez-López and Rodríguez-Ariza, 2011). The orthoptic lecturers have a background in vision sciences, orthoptics and related disciplines and need further education in implementing digital education. The identification of best practices in orthoptics supports lecturers to respond to the individual needs of the students and to provide active learning opportunities. The lecturer training is essential, as one study concluded that students do not take full advantage of the opportunities available in asynchronous (over time) discussions if the e-moderator do not devote considerable time to overseeing the process (Wozniak and Silveira, 2004).

Due to the retrospective nature of this evaluation, this study has a number of limitations, which could be overcome by a prospective study. Questionnaire data was not obtained from two year groups and the student experience from 2014 to 2016 is unknown. The questionnaire results must be interpreted bearing in mind that the platform survey COLLES lacks detailed validity and reliability testing (Baker, 2007). A convenience sampling was used to recruit participants, therefore respondent bias may be an issue, as students who volunteered to participate may be more motivated students. Also, there was the lack of a comparison group for the questionnaire. Students were recruited from only one of the two universities offering the orthoptic degree course in Portugal.

The findings from this study indicate including online learning and a blended learning approach is beneficial for the student experience and academic achievements. We were able to diminish students travelling to the school without compromising achievements. The use of software that automatically collects student data, measures academic performance and progress, provides lecturers and students detailed data, which are important to provide feedback and improve future outcomes. Although the approach used in this study appears to have perceived benefits, a limitation is the assessment of the success in educational outcomes.

The results of this study can be helpful in the preparation of the best approach to support teaching staff and implementing innovative approaches, promoting the use of technology to enhance learning and teaching outcomes. The current model was designed to add on the technology to an existing module. However, a preferred design of a module would be that the technology is design into the teaching. Two major points should be overcome: (1) lecturer’s experience with blended learning approaches and online learning should be increased. Institutional focus should be directed to train lecturers to support the development of pedagogical approaches in developing blended learning and teaching; (2) student’s engagement with online content and classroom activities should be assured. There is a need for a transition between a passive experience to an active experience to engage and encourage students to be successful in an online course. Discussion amongst peers using chat, forums, wikis, workshops and quizzes were not used by the lecturer in these cohorts and the use of these strategies might improve the learning outcomes. The cohort from 2017–2018 is exposed to chats and quizzes, as well as e-assessment tools. Prospective research into the use of online learning and a blended learning approach in orthoptic programmes is warranted.

## Conclusions

In conclusion, the implementation of online learning and a blended learning approach in the research module had a positive impact on student experience, as relevance and reflection were two of the highly rated categories by the participants. Also, the assessment outcomes of the orthoptic students were greater after the second year of the implementation of online learning. However, it was found that more work needs to be done to improve peer support in the online environment, and possibly also in the face-to-face environment. Findings from this study indicate that it may be necessary to provide lecturers with assistance in the design of including online learning and deliver a blended learning approach.

Recommendations for future research related to this study include completion of detailed instrument validity and reliability testing be completed for the online questionnaire, further exploration of strategies for the promotion of peer and a larger prospective study could be implemented to ensure adequate geographical representation and minimization of the issue of respondent bias.

## Disclosures

The author reports no conflicts in relation to this study.

## References

[1] Ally, M. 2004 Foundations of educational theory for online learning In: Anderson, T and Elloumi, F (eds.), Theory and Practice of Online Learning. Athabasca, AB: Athabasca University.

[2] Baker, J. 2007 Constructivist Online Learning Environment Survey In: Baker, J (ed.), Handbook of Research on Electronic Surveys and Measurements, 299–301. IGI Global DOI: 10.4018/978-1-59140-792-8.ch036

[3] Carliner, S. 1999 An Overview of Online Learning. Amherst: Human Resource Development Press.

[4] Dougiamas, M and Taylor, PC. 2002 Interpretive analysis of an internet-based course constructed using a new courseware tool called Moodle. HERDSA 2002 Conference Proceedings The Higher Education Research and Development Society of Australasia.

[5] Ellaway, R and Masters, K. 2008 AMEE Guide 32: e-Learning in medical education Part 1: Learning, teaching and assessment. Med Teach, 30(5): 455–473. DOI: 10.1080/01421590802108331 18576185

[6] Garrison, DR and Kanuka, H. 2004 Blended learning: Uncovering its transformative potential in higher education. Internet High Educ, 7(2): 95–105. DOI: 10.1016/j.iheduc.2004.02.001

[7] Gupta, VK and Gupta, VB. 2016 Using Technology, Bioinformatics and Health Informatics Approaches to Improve Learning Experiences in Optometry Education, Research and Practice. Healthc (Basel, Switzerland), 4(4). DOI: 10.3390/healthcare4040086 PMC5198128 27854266

[8] Iyeyasu, J, Castro, S, Sabbatini, R and Carvalho, K. 2013 The Development and Evaluation of a Distance Learning System in Ophthalmology. Rev Bras Educ Med, 37(1): 96–102. DOI: 10.1590/S0100-55022013000100014

[9] Khan, B. 1997 Web-Based Training. Englewood Cliffs, NJ: Educational Technology Publications.

[10] Khogali, SEO, Davies, DA, Donnan, PT, et al. 2011 Integration of e-learning resources into a medical school curriculum. Med Teach, 33(4): 311–318. DOI: 10.3109/0142159X.2011.540270 21456989

[11] Lewin, LO, Singh, M, Bateman, BL and Glover, PB. 2009 Improving education in primary care: Development of an online curriculum using the blended learning model. BMC Med Educ, 9(1): 33 DOI: 10.1186/1472-6920-9-33 19515243 PMC2702356

[12] Liu, Q, Peng, W, Zhang, F, Hu, R, Li, Y and Yan, W. 2016 The effectiveness of blended learning in health professions: Systematic review and meta-analysis. J Med Internet Res, 18(1): e2 DOI: 10.2196/jmir.4807 26729058 PMC4717286

[13] Lo, CC. 2010 How student satisfaction factors affect perceived learning. J Scholarsh Teach Learn, 10(1): 47–54.

[14] López-Pérez, MV, Pérez-López, MC and Rodríguez-Ariza, L. 2011 Blended learning in higher education: Students’ perceptions and their relation to outcomes. Comput Educ, 56(3): 818–826. DOI: 10.1016/j.compedu.2010.10.023

[15] MacDonald, J. 2008 Developing E-communicators and Collaborators MacDonald, J (ed.), Blended Learning and Online Tutoring: Planning Learner Support and Activity Design, 161–174. Burlington: GOWER.

[16] Makhdoom, N, Khoshhal, KI, Algaidi, S, Heissam, K and Zolaly, MA. 2013 ‘Blended learning’ as an effective teaching and learning strategy in clinical medicine: A comparative cross-sectional university-based study. J Taibah Univ Med Sci, 8(1): 12–17. DOI: 10.1016/j.jtumed.2013.01.002

[17] McBrien, JL, Jones, P and Cheng, R. 2009 Virtual spaces: Employing a synchronous online classroom to facilitate student engagement in online learning. Int Rev Res Open Distance Learn, 10(3): 1–17. DOI: 10.19173/irrodl.v10i3.605

[18] McLaughlin, JE, Gharkholonarehe, N, Khanova, J, Deyo, ZM and Rodgers, JE. 2015 The Impact of Blended Learning on Student Performance in a Cardiovascular Pharmacotherapy Course. Am J Pharm Educ. 79(2): 24 DOI: 10.5688/ajpe79224 25861105 PMC4386745

[19] Morton, CE, Saleh, SN, Smith, SF, et al. 2016 Blended learning: how can we optimise undergraduate student engagement? BMC Med Educ, 16(1): 195 DOI: 10.1186/s12909-016-0716-z 27492157 PMC4973547

[20] Owston, R, York, D and Murtha, S. 2013 Student perceptions and achievement in a university blended learning strategic initiative. Internet High Educ, 18: 38–46. DOI: 10.1016/j.iheduc.2012.12.003

[21] Rowe, M, Frantz, J and Bozalek, V. 2012 The role of blended learning in the clinical education of healthcare students: A systematic review. Med Teach, 34(4): e216–e221. DOI: 10.3109/0142159X.2012.642831 22455712

[22] Salmon, G. 2003 E-Moderating: The Key to Teaching and Learning Online. 2nd ed London: RoutledgeFalmer.

[23] Secomb, J. 2008 A systematic review of peer teaching and learning in clinical education. J Clin Nurs, 17(6): 703–716. DOI: 10.1111/j.1365-2702.2007.01954.x 18047577

[24] Sthapornnanon, N, Sakulbumrungsil, R, Anuchai, T and Watcharadamrongkun, S. 2009 Social Constructivist Learning Environment in an Online Professional Practice Course. Am J Pharm Educ, 73(1): 1–8. DOI: 10.5688/aj730110 19513147 PMC2690880

[25] Syed-Mohamad, S-M, Pardi, K-W, Zainal, N-A and Ismail, Z. 2006 Expanding Nursing Education through e-Learning: A Case Study in Malaysia. In: Park, H-A, Murray, PJ and Delaney, C (eds.), Consumer-Centered Computer-Supported Care for Healthy People: Proceedings of NI2006, The 9th International Congress of Nursing Informatics, 186–189. Netherlands: IOS Press.

[26] Taylor, P and Maor, D. 2000 Assessing the efficacy of online teaching with the Constructivist Online Learning Environment Survey. In: Herrmann, A and Kulski, MM (eds.), Flexible Futures in Tertiary Teaching: Proceedings of the 9th Annual Teaching Learning Forum Perth: Curtin University of Technology http://lsn.curtin.edu.au/tlf/tlf2000/taylor.html.

[27] The University of Sheffield. 2016 Our University, Our Future, Our Plan. Sheffield https://www.sheffield.ac.uk/ourplan/wp-content/uploads/2015/12/TUOS-Strategic-Plan.pdf.

[28] University of Oxford. 2017 Digital strategy University of Oxford https://www.ox.ac.uk/about/organisation/digital-strategy?wssl=1 Published 2017. Accessed July 12, 2017.

[29] Wozniak, H. 2006 Online discussions: Improving the quality of the student experience In: Tulloch, M, Relf, S and Uys, P (eds.), Breaking down Boundaries: International Experience in Open, Distance and Flexible Education – Selected Papers, 170–179. Charles Sturt University, Bathurst: Open and Distance Learning Association of Australia.

[30] Wozniak, H and Silveira, S. 2004 Online discussions: Promoting effective student to student interaction. In: Atkinson, CM, Jonas-Dwyer, D and Phillips, R (eds.), Beyond the Comfort Zone: Proceedings of the 21st ASCILITE Conference, 956–960. Perth.

